# Supplementary figures and images for: Revisiting suicide rate during wartime: Evidence from the Sri Lankan civil war
Source: PLoS One. 2020 Oct 28;15(10):e0240487. doi: 10.1371/journal.pone.0240487 (PMC7592752; doi:10.1371/journal.pone.0240487)

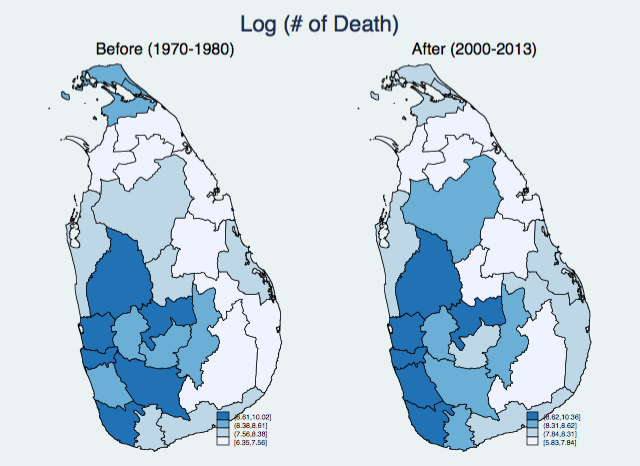

Supplement: S1 Fig — The figures show the average of the log (# of death) from 1970 to 1980 (Panel A) and from 2000 to 2013 (Panel B). (TIF) [file pone.0240487.s001.tif]

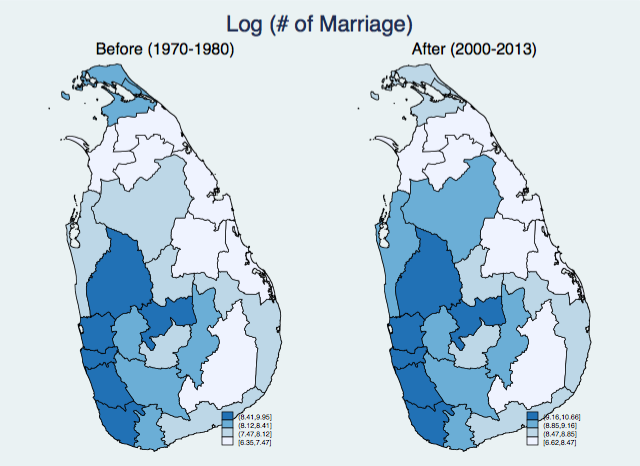

Supplement: S2 Fig — The figures show the average of the log (# of marriage) from 1970 to 1980 (Panel A) and from 2000 to 2013 (Panel B). (TIF) [file pone.0240487.s002.tif]

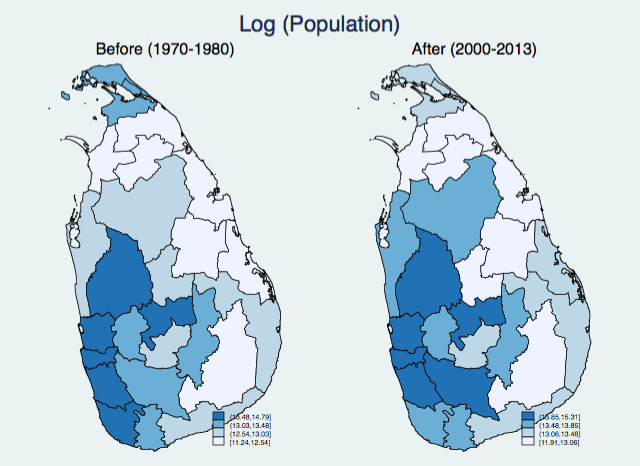

Supplement: S3 Fig — The figures show the average of the log (population) from 1970 to 1980 (Panel A) and from 2000 to 2013 (Panel B). (TIF) [file pone.0240487.s003.tif]

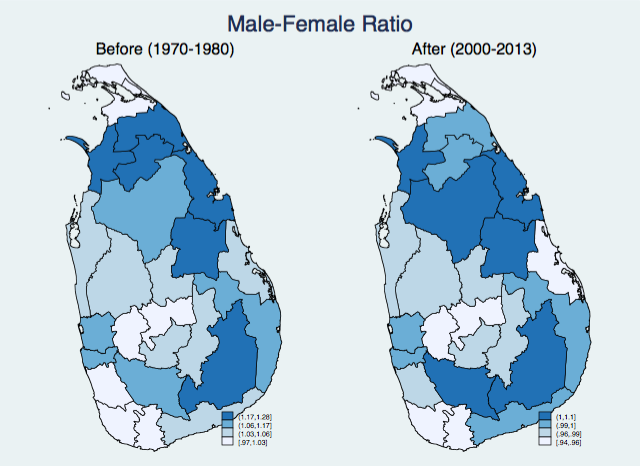

Supplement: S4 Fig — The figures show the average of the male-female Ratio from 1970 to 1980 (Panel A) and from 2000 to 2013 (Panel B). (TIF) [file pone.0240487.s004.tif]

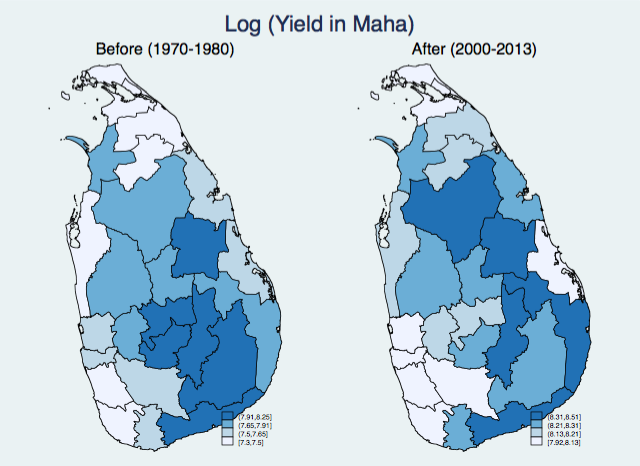

Supplement: S5 Fig — The figures show the average of the log (yield in Maha) from 1970 to 1980 (Panel A) and from 2000 to 2013 (Panel B). (TIF) [file pone.0240487.s005.tif]

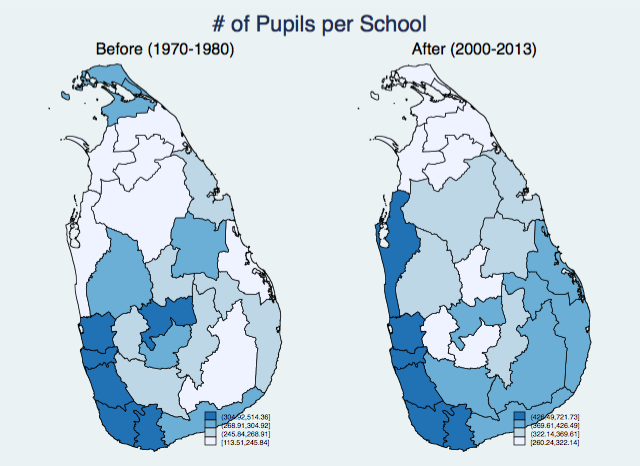

Supplement: S6 Fig — The figures show the average of the # of pupils per school from 1970 to 1980 (Panel A) and from 2000 to 2013 (Panel B). (TIF) [file pone.0240487.s006.tif]

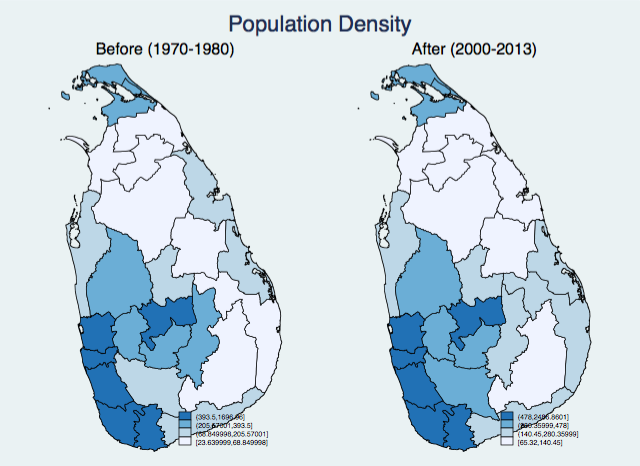

Supplement: S7 Fig — The figures show the average of the population density from 1970 to 1980 (Panel A) and from 2000 to 2013 (Panel B). (TIF) [file pone.0240487.s007.tif]

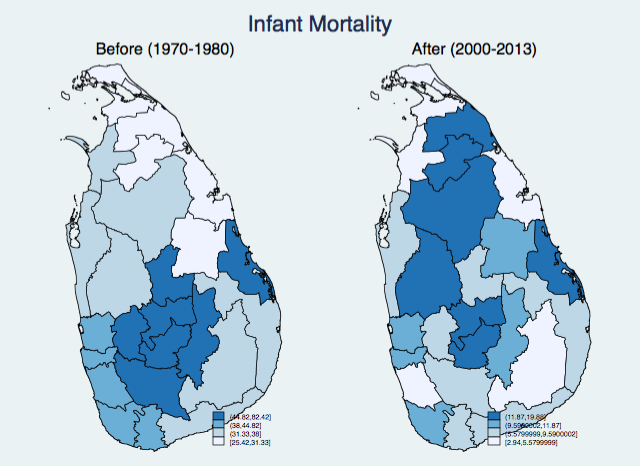

Supplement: S8 Fig — The figures show the average of the infant mortality from 1970 to 1980 (Panel A) and from 2000 to 2013 (Panel B). (TIF) [file pone.0240487.s008.tif]
