# Supplementary material for: Revisiting suicide rate during wartime: Evidence from the Sri Lankan civil war
Source: PLoS One. 2020 Oct 28;15(10):e0240487. doi: 10.1371/journal.pone.0240487 (PMC7592752; doi:10.1371/journal.pone.0240487)
Supplement: S1 File — (ZIP) [file pone.0240487.s009.zip › replication/readme.rtf]

This is the supplementary material for "Revisiting Suicide Rate during Wartime: Evidence from the Sri Lankan Civil War,” by Takeshi Aida.This folder includes:suicide_all_rev.dta: STATA dta file containing the data used for the analysissuicide_srilanka.do: STATA do file to replicate the resultdistrict_adjacent22.csv: Adjacency matrix of administrative districtssrilanka_data and srilanka_coordinates.dta: Boundary of administrative districts (converted to STATA data files)violence.csv: Statistics of grievous hurts and homicidesged181.csv: Information on conflicts downloaded from the UCDP webpage (https://ucdp.uu.se/)
