# Supplementary material for: Revisiting suicide rate during wartime: Evidence from the Sri Lankan civil war
Source: PLoS One. 2020 Oct 28;15(10):e0240487. doi: 10.1371/journal.pone.0240487 (PMC7592752; doi:10.1371/journal.pone.0240487)
Supplement: Attachment — Submitted filename: response_letter.pdf [file pone.0240487.s010.pdf]

Response Letter

PONE-D-20-10169
Revisiting suicide rate during wartime: Evidence from the Sri Lankan civil war
PLOS ONE

[Comments from the editor]

Thank you for submitting this interesting work. Both reviewers found the topic important and of interest. Both also found significant limitations in the theoretical approach and evaluation of local cultural factors. I concur with Reviewer 2 that a major revision is appropriate. Please address the cultural factors, perhaps consulting Sri Lankan' sources on local views toward the civil war and suicide. Additional theoretical approaches should also be examined. This work may actually demonstrate limitations in Durkheim's theory, rather than support. That may be a contribution of this study. In addition, please address issues with the data. Table 2 requires dates for the pre-war and war periods. Also, why not include post-war data? Typically, we provide data at two decimal places, rather than three. Tables 4-8 require more clarification. Mention the type of analysis in the title, and more details of the variables, what do the numbers represent? If these are regression models, they require R-squared values and standardized beta values for better interpretation of the total model and individual predictors. Overall, the analyses and results require better elaboration and further interpretation.

[Reply]

Thank you for your kind consideration and constructive comments. I hope the current version conforms to the standards of your journal.

- In accordance with the suggestions from you and Reviewer #2, I have included a discussion on the cultural factors surrounding the stigmatization of suicide in Sri Lanka.
- I want to thank you for this very important comment. Taking heed of your and the reviewers' advice, I have elaborated on Durkheim's theory and how civil wars can lead to lower suicide rates. Specifically, several studies in comparative politics have shown that civil wars harden ethnic identities. Thus, civil wars may boost the level of integration, although its body comprises ethnic groups rather than a nation, and may lower suicide rates.
- I have added the year in Table 2.

- I found a new data source and expanded my data to include the post-war period (years 2010 and 2013). Although it is still limited, it provides us some clues about the suicide rate in the post-war period. It is also mentioned in the discussion section that further expansion of the data is an important work that still remains unfinished.
- The data is now shown up to two decimal places.
- I have added a more detailed interpretation of the results shown in Tables 4–10.
- I have mentioned the type of analysis in the title of the tables and added the units of each variable in Table 3 as well as in the main text.
- R-squares are in the tables of the regression results (Tables 4–10). As for standardized beta values, I agree that they make the comparison of the coefficients easier in general. However, our primary variable of interest (that is, a cross-term of the wartime and contested area dummies) is a dummy variable. Therefore, the point estimate is directly translated into the change in the suicide rate compared to the non-contested area in the peacetime period. For this reason, I am worried that standardized beta values might make the interpretation more difficult. Additionally, I have not interpreted other coefficients because they are just control variables, and thus, this should not be interpreted as being causal. Therefore, I have kept the variables unstandardized and have mentioned these issues in the text. However, if you think that standardized beta values are better for the control variables, I would be happy to make changes accordingly.

[Comments from Reviewer #1]

The paper addresses an important issue, but I do not think the Sri Lankan civil war (1983 - 2009) provides suitable case study of the issue. This was a separatist was between LTTE (a group of radical youth from the second largest ethnic group, Tamils, of the country) and the government). There was no broad community support for the LTTE among in the Tamil community in the contested districts. Put simply, during this period certainly there was no 'increased social integration' that would have led to fewer suicide cases in these districts as postulated by Durkhein (1887).

Given this fundamental flaw, I do not which to make detailed comments on this paper.

[Reply]

Thank you for pointing out a very critical issue. It is true that the Sri Lankan Civil War was fought between a group of separatists (LTTE) and the government, which is different from the wars discussed by Durkheim.

In order to incorporate the suggestions in your comment, as well as the one from Reviewer #2, I have rewritten the introduction. In the current version, I have elaborated on Durkheim's theory and explained why it may be applicable to civil wars. Specifically, several studies in comparative politics have shown that civil wars harden ethnic identities. Thus, it is possible that civil wars enhance the level of integration, although its body consists of ethnic groups rather than a nation, and lead to lower suicide rates. I have mentioned this as an important issue remaining to be addressed in the introduction.

[Comments from Reviewer #2]

The article under consideration investigates the relationship between war and suicide at the local-level using Sri Lanka as a case study. I find the topic important, and was impressed by the work the author did to cope with limited data on the topic. That said, I do have several concerns that I summarize below.

• The premise is the work of Durkheim, who argues that war increases social integration, thus leading to fewer suicides. The issue here is that the author does not really bring this logic into the article, so the reader is left wondering what the debate is about. I would like to see a clear theoretical discussion about how this logic works, and why specifically war is able to increase social integration.

[Reply]

I am much obliged for your very detailed and constructive comments. I have incorporated your suggestions to the best of my ability. I firmly believe that the manuscript has improved significantly.

In response to your observation, I have elaborated on Durkheim's theory in the introduction. Specifically, he argues that wars "sharpen collective feelings, stimulate the party spirit and the national one and, by concentrating activities towards a single end, achieve, at least for a time, greater integration of society." (Durkheim, 1897 [1]) Thus, it is understood that wars increase social integration, which leads to lower suicide rates.

It is worth noting that war has changed dramatically since 1897 (when Durkheim published this piece), so it is important for the author the explain the mechanisms by which the war in Sri Lanka might increase social integration. I think this argument is clear in the context of interstate wars, but not civil wars – especially one as long-lasting as Sri Lanka.

[Reply]

Thank you for pointing out a very critical issue. It is true that Durkheim considered inter-state wars in his argument while the war in Sri Lanka was a civil conflict, which was fought between the LTTE and the government.

In accordance with your comment, as well as the one from Reviewer #1, I have rewritten the introduction. Specifically, several comparative political studies have shown that civil wars

harden ethnic identities. Thus, it is possible that civil wars increase the level of integration, though its body encompasses ethnic groups rather than a nation, and lower suicide rates. I have mentioned this as an important issue remaining to be addressed in the introduction.

• The author also notes that the "other side" argues that confounding factors are driving this relationship, but fails to elaborate. What exactly does this side of the literature argue, and what confounding factors are seen as being most significant?

[Reply]
I have elaborated on the omitted confounding factors discussed in the previous studies in the revised introduction. Specifically, they are mainly economic conditions and time-trend. As for the economic conditions, I included the yield of paddy in the primary cropping season. Unfortunately, I could not include the unemployment rate because of the non-availability of data. However, I have discussed why the unemployment rate is not a severe concern in this study. As for the trend effect, the difference-in-difference approach with year fixed effects is a more flexible way to control it than including the linear trend effect. For these reasons, these factors are not so critical as to change the findings of this study.

• In my view, the introduction could be improved by being explicit about the importance of the study. The author should look to frame their contribution in the larger body of work that looks at the consequences of civil wars, and the fact that these consequences linger long after the guns fall silent. Or, alternatively, the author could cite some literature that speaks to the societal and personal costs of suicide. But, in any case, some work needs to be done to improve this.

[Reply]
I appreciate this extremely significant recommendation. As mentioned above, I have extensively rewritten the introduction following your advice. In the revised version, I have explained that the applicability of Durkheim's theory in the context of civil wars is an important issue remaining to be addressed. In the discussion, I have mentioned that the existing studies have mainly focused on the long-lasting effect of civil wars on physique and diseases, but the issue of wartime suicides has been largely unexplored.

• Because the war in Sri Lanka was so prolonged, there is a need to discuss the evolution of

the war. Specifically, I would like to see some data (or a discussion if data is not possible) that lays out patterns of violence over time. The empirical work here only looks at a brief snapshot of the war (which was during the final phase), so it would be useful to see how this period compares to others.

[Reply]

I have added a figure (Fig 4) that demonstrates the pattern of violence (the numbers of grievous hurts and homicides) over time. Except for the outstanding increase in the number of homicides in 1988 and 1989, which may reflect the intensity of the war, there is no clear trend in the pattern of violence during the war. Although data during this period should be interpreted with caution, this graph shows that our sample period (2000–2009) is not a peculiar time period during the war. I have added this discussion to the main text.

• A discussion of how the suicide rate was calculated in this case would be helpful. It is very difficult to get information during wartime, even battle deaths. This implies that any suicide rates reported during wartime should be interpreted with extreme caution.

[Reply]

Although the suicide rates are gathered from several sources, all of them were originally sourced from the Registrar General's Department. They collect data via a registrar in each registration division, each of which is further divided into administrative districts. I have added this issue to the text.

The suicide rate in Sri Lanka is known to be just about as reliable as that from many developed countries (Kearney and Miller, 1988 [21]). That said, I completely agree that the suicide rate reported during the war should be interpreted with caution. I have mentioned this in the main text. For this reason, I conducted a robustness check by dropping the districts where the census in 2002 was not conducted due to the high intensity of war, assuming that the reliability of the statistics is not high in these districts (Table 6). The estimates are still significantly negative, suggesting the robustness of the accuracy of data, albeit not perfectly.

How heavily is suicide stigmatized in Sri Lanka? Is there an incentive to add suicides to fatalities via the war rather than acknowledge suicide? How exactly does this process play out? I know some of these questions may not be answerable, but this has important implications for the negative relationship that is uncovered. I would like to see the author at

least address this potential issue.

[Reply]
Although it is difficult to show the extent to which suicide is stigmatized in Sri Lanka, Marecek (1998) [24] mentions that the suicide of a family member exposes the presence of family problems and might diminish the marital prospects of children. In this sense, it is difficult to strictly rule out the possibility that there is an incentive to add suicide to the fatalities via the war. However, according to the Criminal Procedure Code, unnatural deaths, including suicides, are subject to inspection by a coroner before the cause of death is concluded (Fernando et al. 2003 [25]). Therefore, it is unlikely that fatalities of the war are conflated with suicide, although this possibility cannot be strictly ruled out. I have mentioned this issue in the main text.

• Empirically, I have two concerns. First, I am not entirely sure where the control variables come from or how they are distributed across space. Because the study is done at the local-level it is not enough to show the aggregate distribution, the author must also show local variation. Creating some maps would be great here.

[Reply]
I apologize for not mentioning this clearly. All the control variables have been sourced from *Statistical Abstract*, an official annual statistical report issued by the Department of Census and Statistics. Following your advice, I have created heat maps of the control variables to show variations at the district level. These maps are included in the supporting materials.

Second, there is notable spatial clustering in Figure 2. Has the author ruled out spatial dependence? Or, is there a need to account for diffusion in the models?

[Reply]
Thank you for this suggestion. I have added paragraphs about the analysis of spatial dependence. Specifically, I estimated a spatial autoregressive model and the spatial difference-in-difference model to control possible spillover effects from the contested area to the non-contested area (Tables 8 and 9). However, in either approach, the reduction in the suicide rate in the contested area during the war is virtually unaffected, suggesting the robustness of the findings.
