# Supplementary material for: Revisiting suicide rate during wartime: Evidence from the Sri Lankan civil war
Source: PLoS One. 2020 Oct 28;15(10):e0240487. doi: 10.1371/journal.pone.0240487 (PMC7592752; doi:10.1371/journal.pone.0240487)
Supplement: Attachment — Submitted filename: Reply to Comments.pdf [file pone.0240487.s011.pdf]

Response Letter

PONE-D-20-10169

Revisiting suicide rate during wartime: Evidence from the Sri Lankan civil war

Thank you very much, once again, for your detailed comments. I have incorporated your comments as indicated below.

Line 22: “to 43..” should be “to a 43..”

[Reply] Modified

Line 39: “Indeed” should be removed or changed. Also, this is not a paragraph. Paragraphs should be 3 or more sentences, consider combining some sections into full paragraphs.

[Reply] I have changed the quotation style and combined the sentences that followed with the previous paragraph. I have also removed the word “Indeed.”

Line 45: “harden ethnic minorities” this is not clear, needs clarification

[Reply] The original expression was “harden ethnic identities” and not “harden ethnic minorities.” Hence, I have retained this expression as it is.

Line 55: “the second important issue” this is not the best writing style, and needs clarification. Please remind readers of the issue you are writing about.

[Reply] I have changed “the first important issue” and “the second important issue” to “one of the most important issues” and “another important issue,” respectively.

Line 63: Please state the years of the Sri Lankan war, with citation, and also clarify “the Sri Lankan suicide rate.”

[Reply] The years in which the war took place were stated right after the sentence, and I have added the citation for this. I have also modified “the Sri Lankan suicide rate” into “the annual suicide rate in Sri Lanka.”

Line 83 “more rigorous statistical analysis” More rigorous than what? Also, “exploiting”

is not the best word here.

[Reply] I have added “than previous studies.” I have also modified “exploiting” into “estimating.”

Line 89: It isn’t clear why this study is “more definitive.” Better to just emphasize that you have included additional factors etc.

[Reply] I have added “in the sense that it provides robust evidence to possible confounding factors.” Please note that the difference-in-differences approach is different from the approaches that simply include additional factors in the regression model.

Line 94: “the district level” that is not clear, specific that this relates to SL, and what districts are.

[Reply] I have added an explanation clarifying that, in Sri Lanka, the district is the second-level administrative division after the province.

Line 106: The births/deaths act requires a citation and also mention that this is for SL.

[Reply] I have added a citation and mentioned that this is for Sri Lanka.

Line 112: “That being said” is informal, and also not appropriate for beginning a paragraph, should be deleted.

[Reply] Modified

Line 134: “Fig 1” should be spelled out in full in text. Also correct for other figure mentions.

[Reply] Modified

Table 1 – this is very brief and does not require a table. Consider adding additional data or just writing out in text.

[Reply] I have deleted Table 1 and added some text from Line 163.

Line 178: clarify what “the contrast” refers to.

[Reply] I have clarified that it is the contrast between the contested and the non-contested districts.

> Table 3: delete "#" this can be changed to "deaths" or "total deaths" for example. It is unclear what this symbol means under "Units"

[Reply] Modified

> Table 5 – "R-squared" should be R2 and this does not require a lead "0".

[Reply] Modified

> Line 330: "the third possible.." Again, this is not clear, specify what this refers to, and again later.

[Reply] I have modified "the possible concern" to "possible concern about the robustness of the findings."

> Line 387: as with the rest of the manuscript, this should be in past tense.

[Reply] Modified

> Line 474: "decrease in suicide rate" this needs to be clearer, annual suicide rate in SL? Specify this point.

[Reply] I have specified that it is the annual suicide rate in Sri Lanka.

> Line 503: "It is known that.." This is informal and should be revised.

[Reply] Modified
